# A New Approach to Investigate the Association between Brain Functional Connectivity and Disease Characteristics of Attention-Deficit/Hyperactivity Disorder: Topological Neuroimaging Data Analysis

**DOI:** 10.1371/journal.pone.0137296

**Published:** 2015-09-09

**Authors:** Sunghyon Kyeong, Seonjeong Park, Keun-Ah Cheon, Jae-Jin Kim, Dong-Ho Song, Eunjoo Kim

**Affiliations:** 1 Department of Psychiatry and Institute of Behavioral Science in Medicine, Yonsei University College of Medicine, Seoul, Republic of Korea; 2 Brain Korea 21 PLUS Project for Medical Science, Yonsei University, Seoul, Republic of Korea; 3 Division of Mathematical Models, National Institute for Mathematical Sciences, Daejeon, Republic of Korea; Institute of Psychology, Chinese Academy of Sciences, CHINA

## Abstract

**Background:**

Attention-deficit/hyperactivity disorder (ADHD) is currently diagnosed by a diagnostic interview, mainly based on subjective reports from parents or teachers. It is necessary to develop methods that rely on objectively measureable neurobiological data to assess brain-behavior relationship in patients with ADHD. We investigated the application of a topological data analysis tool, *Mapper*, to analyze the brain functional connectivity data from ADHD patients.

**Methods:**

To quantify the disease severity using the neuroimaging data, the decomposition of individual functional networks into normal and disease components by the healthy state model (HSM) was performed, and the magnitude of the disease component (MDC) was computed. Topological data analysis using *Mapper* was performed to distinguish children with ADHD (*n* = 196) from typically developing controls (TDC) (*n* = 214).

**Results:**

In the topological data analysis, the partial clustering results of patients with ADHD and normal subjects were shown in a chain-like graph. In the correlation analysis, the MDC showed a significant increase with lower intelligence scores in TDC. We also found that the rates of comorbidity in ADHD significantly increased when the deviation of the functional connectivity from HSM was large. In addition, a significant correlation between ADHD symptom severity and MDC was found in part of the dataset.

**Conclusions:**

The application of HSM and topological data analysis methods in assessing the brain functional connectivity seem to be promising tools to quantify ADHD symptom severity and to reveal the hidden relationship between clinical phenotypic variables and brain connectivity.

## Introduction

Attention-deficit/hyperactivity disorder (ADHD) is the most common neurodevelopmental disorder of childhood, affecting at least 5% of school-age children worldwide [1]. Children with ADHD usually experience symptoms of inattention, impulsivity, and hyperactivity. ADHD is also associated with impairments in academic, social, and family functioning and is commonly accompanied by a range of comorbid psychiatric disorders [2].

Owing to the limited understanding of the biological underpinnings of mental disorders, ADHD is currently diagnosed using the criteria from the Diagnostic and Statistical Manual of Mental Disorders (DSM) [3] rather than by objective neurobiological evidence [4–7]. In fact, symptoms are usually reported by parents or teachers based on their inherently subjective observations. Moreover, diagnosing ADHD can be challenging because the line between normal behaviors typically observed in children and ADHD symptoms is somewhat arbitrary [8]. Diagnosis of ADHD is further complicated by the presence of comorbid conditions, including learning disabilities, oppositional defiant disorder (ODD), anxiety disorders, and mood disorders [8]. As a result, the rates of ADHD reported in epidemiological studies are often variable and sometimes overestimated [8], and misdiagnoses are reported as ranging approximately from 10% to 30% [9]. Therefore, it would be highly desirable to develop objective methods that rely on objectively measurable data.

With this background, the interest in neurobiological markers of ADHD has grown substantially in recent years [10–14]. In particular, the feasibility of predicting disease states using data from structural and functional magnetic resonance imaging (MRI) has attracted increasing attention in the field and has shed light on the development of imaging-based diagnostic tools to complement the clinicians’ diagnosis [15–24]. For example, the recent competition announced by the ADHD-200 Consortium aimed to develop various types of supervised or modified versions of classical learning algorithms to distinguish ADHD from typically developing children (TDC) using resting state functional imaging datasets from large samples [6,25–27]. However, the average prediction accuracy was 49.8% (range 37.4–60.5%), and the team that generated the highest score only used phenotypic data of age, sex, IQ, and handedness, without even using imaging data [6]. Therefore, the question of whether imaging-based features are better predictors of ADHD than demographic features became a debated issue in the imaging community [6,27]. So far, imaging data do not seem to provide diagnostic benefits and potential MRI-based biomarkers, which are useful for a direct diagnostic decision and a measure of disease severity, are rarely attained [4,28,29].

To overcome this limitation, new methods for assessing disease characteristics from the neuroimaging data need to be developed and the phenotypic associations in high-dimensional brain connectivity data need to be identified. For this purpose, we are investigating the application of mathematical models to the analysis of brain functional connectivity data. The recently developed topological data analysis tool, called *Mapper*, is widely used in analyzing high dimensional behavioral [30], clinical [31,32], and biological [33] datasets. *Mapper* is a mathematical framework and was developed in the area of applied topology to identify shape characteristics of datasets based on the distance between data points along a pre-assigned filter function [34]. Usually, filter functions for the disease-specific data analysis are provided by the healthy state model (HSM), which was first introduced in microarray data analysis [32]. The HSM essentially unravels the data according to the extent of overall deviation from a healthy (or normal) state, and provides a means to define the guiding filter functions. *Mapper*, guided by the filter and distance functions, approximately collapses the data into a simple and low dimensional shape. *Mapper* was successfully applied to genomic expression data from diseased tissue, and classifying breast cancer [31] and diabetes subtypes [34].

In this study, we present the topological analysis tool, *Mapper*, in combination with HSM and their application to functional neuroimaging data. We investigated the association between the disease components analyzed by *Mapper* and HSM with clinical phenotypes such as IQ, symptom severity and the comorbidity rate of ADHD to test whether brain functional connectivity patterns are related to differences in these phenotypic variables of interest.

## Methods and Materials

### Datasets

The preprocessed resting state fMRI data was obtained from the ADHD-200 Consortium website (http://fcon_1000.projects.nitrc.org/indi/adhd200). We selected datasets from New York University (NYU) and Peking University (PU) for our study because these two institutes have the largest data samples among the ADHD-200 database and these datasets include equal amount of patients with ADHD and TDC. The NYU dataset includes 98 TDC and 118 children with ADHD. The PU dataset includes 116 TDC and 78 children with ADHD. Psychiatric diagnoses, including comorbidity information, were established through psychiatric interviews with experienced child psychiatrists using the Schedule of Affective Disorders and Schizophrenia for Children-Present and Lifetime Version administered to parents and children (NYU and PU) and the Conners’ Parent Rating Scale-Revised, Long Version (NYU) or the ADHD Rating Scale IV (PU). Symptom severity such as inattention and hyperactivity/impulsivity and the ADHD index, which is an overall measure of ADHD symptom severity, were rated by parents. Intelligence was evaluated with the Wechsler Abbreviated Scale of Intelligence (NYU) or the Wechsler Intelligence Scale for Chinese Children-Revised (PU). The details for the phenotypic and clinical variables are described elsewhere [35].

### Preprocessing

**Fig 1:**
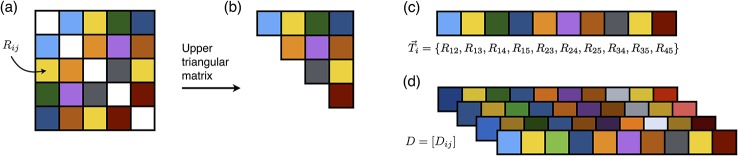
Schematic procedures of the functional network construction. (**A**) Functional network matrix, **[*R***_***ij***_ **]**, for a subject. (**B**) Upper triangular matrix of **[*R***_***ij***_ **]**. (**C**) Vectorization of the upper triangular matrix. (**D**) Stacking ***T***_***i***_ for all subjects to construct ***D***, where the vector ***T***_***i***_ is the *i*-th row vector of ***D* = [*D***_***ij***_ **]**.

Briefly, for the construction of the functional network, we used the extracted time courses from the Athena preprocessed data. A detailed description of the preprocessing steps can be found in the Supporting Information as well as on the Athena website. The filtered time course files, ADHD200_AAL_TCs_filtfix.tar.gz, can be downloaded from the ADHD-200 Preprocessed Data website. The functional network of each subject (*R*_*ij*_) was then computed by Pearson’s correlation coefficients between the time courses of *i*-th and *j*-th regions of interest (ROIs). The upper triangular part of the functional connectivity matrix for each subject was extracted and vectorized as following:

$$T_{i}=\left\{R_{12},R_{13},\ldots \;,R_{1n},R_{23},\ldots \;,R_{2n},\ldots \;,R_{n-1,n}\right\},$$ (1)

where *i* is the subject index, *n* is the number of ROIs, and the dimension of *T*_*i*_ is *m* = *n*(*n* − 1)/2. Here, we called the vectorized functional connectivity data as functional connectivity vector *T*_*i*_. Finally, the functional network dataset, *D* = [*D*_*ij*_], can be obtained as illustrated in **Fig 1**, where *i* represents the subject index, *j* represents the *j*-th elements of *T*_*i*_, and the vector *T*_*i*_ is the *i*-th row vector of *D* = [*D*_*ij*_].

### Healthy state model (HSM)

The functional connectivity vector *T*_*i*_ of each subject as described in Eq (1) can be decomposed into the normal component and the disease component by HSM [32] as follows:

$$T_{i}=T_{i}^{Nc}+T_{i}^{Dc},$$ (2)

where the normal component (Nc) of data mimics HSM. The detailed description about HSM can be found in the SI. Then, the residual of the fit to HSM becomes the disease component as follows:

$$T_{i}^{Dc}=T_{i}-T_{i}^{Nc}.$$ (3)

Finally, the magnitude of the disease component for each subject can be obtained using *L*_2_-norm as follows:

$$\|T_{i}^{Dc}\|=\sqrt{\sum_{\forall_{uv}}{\left|R_{uv}^{Dc}\right|}^{2}},$$ (4)

where $R_{uv}^{Dc}$ is a residual of the disease component in the individual functional network. The *L*_2_-norm of the disease component measures the overall amount of deviation from the HSM.

### Topological data analysis

In this study, the topological data analysis was used to extract a geometric shape from the relationships among subjects by using a new technology named “partial clustering”. Initially, *Mapper*, a tool for topological data analysis [31,34] was introduced in the neuroimaging society. The first step for analyzing neuroimaging data using *Mapper* is to define distance and filter functions. The use of distance function is to measure dissimilarity between disease components of the individual functional connectivity vector. Usually, the correlation distance is used as a distance function:

$$d\left(T_{u}^{Dc},T_{v}^{Dc}\right)=1-r\left(T_{u}^{Dc},T_{v}^{Dc}\right),$$ (5)

where *r*(*X*, *Y*) measures correlation coefficients between two vectors: *X* and *Y*.

The essential role of the filter function is to collapse high-dimensional functional network data to a single data point and to capture a neurobiologically meaningful characteristic of the data [31]. In the current study, the filter function measured the magnitude of the disease component in the functional network data. In general, the value of the filter function becomes larger when a large number of functional connections deviate by a large extent from the HSM either in a positive or negative direction. The second step for the topological data analysis is to define the clustering method. We chose the single-linkage method that was widely used in the topological data analysis. A detailed description of these particular clustering procedures can be found in the references [33,36]. The last step is to visualize the resulting topology using a graph (**Fig C in S1 File**). In the resulting graph, each node is a subset of subjects, and edges connect similar nodes. The color of each node encodes the value of the filter function averaged across all the data points to the node, with blue representing a low value and red denoting a large value.

### Statistical analysis

First, group differences in the values of the filter function, which represent the magnitude of the disease component, were examined by a one-way analysis of variance (ANOVA). Second, Pearson’s correlation coefficients between the values of the filter function and clinical phenotypic variables were evaluated to find the significant relationships between these measures. Third, for the value of the filter function, we conducted analysis of the receiver operating characteristics including the estimation of sensitivity and specificity. Fourth, the correlation analysis was conducted to reveal a relationship between a psychiatric comorbidity and resulting topology. For this analysis, we calculated Pearson’s correlation coefficients between the ratios of the subject with psychiatric comorbidity in each node in the resulting topology and the node index, where the node index represents the node number with lower (higher) index indicates a subset of subjects having a lower (higher) value of the filter function.

## Results

### Demographic variables and clinical measures

Some differences existed in the clinical characteristics between participants from NYU and PU (**Table 1**). There was no significant difference in age between the TDC and ADHD group, but the proportion of males was higher in the ADHD sample compared to the TDC sample. In the dataset, several subjects did not have scores from clinical measures and were excluded from the correlation analysis (**Table 2**). Scores of the ADHD index were significantly lower for PU than for NYU participants (*p* < 0.0005, **Table A in S1 File**), which likely reflect differences between the ADHD Rating Scale IV (PU) and the Conners’ Parent Rating Scale-Revised, Long Version (NYU). In addition, we computed the ratio of comorbidity in patients with ADHD for the NYU and PU datasets. In the NYU dataset, 36% (42 of 118) of patients with ADHD had the following comorbid psychiatric symptoms: anxiety disorder (15 patients), depressive disorder (8 patients), learning disorder (LD, 6 patients), ODD (6 patients), and other disorders (7 patients). In the PU dataset, 53% (41 of 78) of patients with ADHD had the following comorbid psychiatric symptoms: ODD (25 patients), LD (7 patients), tics (6 patients), conduct disorder (3 patients). Due to this substantial difference in clinical characteristics of each dataset, analyses were conducted separately for the NYU and PU datasets.

**Table 1:** Demographic variables and ADHD diagnoses.

Data Set	TDC		ADHD		ADHD Diagnosis		
	Sex	Age	Sex	Age	Combined	Inattentive	Hyperactive
	(M/F)	Mean (SD)	(M/F)	Mean (SD)			
NYU	47/51	12.2 (3.1)	93/25	11.3 (2.7)	73	43	2
PU	71/45	11.7 (1.7)	73/5	12.4 (2.0)	29	49	0

ADHD, attention-deficit/hyperactivity disorder; F, female; M, male; NYU, New York University Child Study Center; PU, Peking University; SD, standard deviation; TDC, typically developing children

**Table 2:** Correlations between values of the filter function and clinical phenotypes (symptom severity and intelligence).

Clinical phenotype	TDC group		ADHD group	
	NYU (*n* = 98)	PU (*n* = 116)	NYU (*n* = 118)	PU (*n* = 78)
Symptom Severity				
Missing data, *n*	2	15	2	7
ADHD Score	-0.10 (0.3404)	0.08 (0.4395)	0.03 (0.7897)	0.23 (0.0488)*
Inattentive Score	-0.11 (0.3006)	0.12 (0.2346)	-0.04 (0.7015)	0.22 (0.0655)
Hyperactivity/Impulsivity	-0.05 (0.5956)	0.01 (0.9363)	0.04 (0.6993)	0.20 (0.0986)
Intelligence Scale				
Missing data, *n*	7	1	3	0
Full-Scale IQ	-0.34 (0.0009)**	-0.19 (0.0458)*	-0.06 (0.5513)	-0.17 (0.1273)
Performance IQ	-0.32 (0.0017)**	-0.18 (0.0497)*	-0.01 (0.9326)	-0.05 (0.6811)
Verbal IQ	-0.28 (0.0067)*	-0.14 (0.1355)	-0.08 (0.3940)	-0.20 (0.0780)

Values are Pearson’s correlation coefficients (plus corresponding *p*-values).

The statistically significant thresholds are labeled as *P < 0.05

**P < 0.005.

### Distribution of disease component

**Fig 2:**
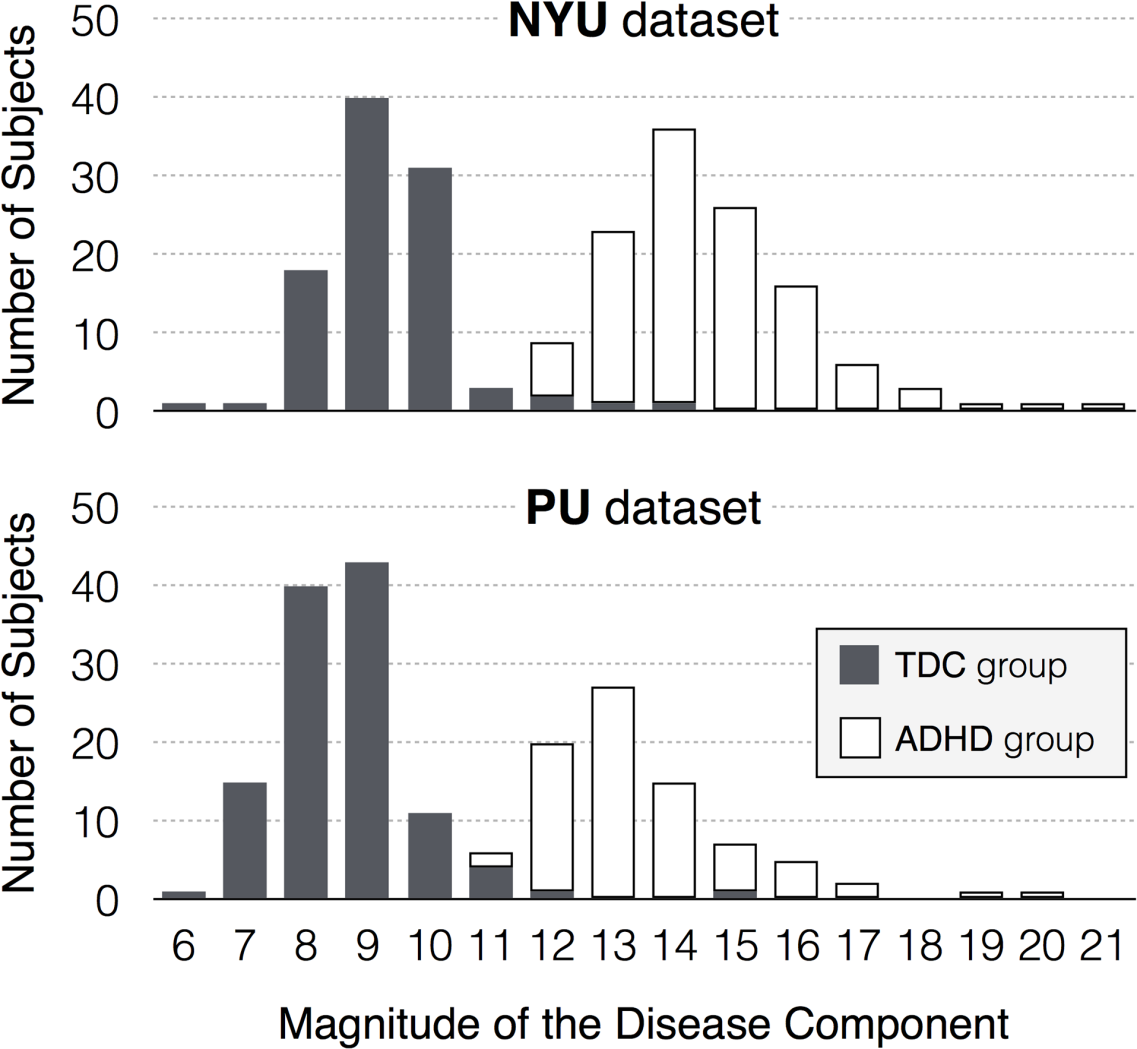
Distribution of the magnitude of the disease component (or the values of the filter function) for each group: the TDC group (gray bars) and the ADHD group (white bars).

The filter function successfully measured a magnitude of the disease component; the subjects with smaller values of the filter function, which represented the smaller magnitude of the disease component, were mostly in the TDC group while the subjects with larger values of the filter function were mostly patients with ADHD. **Fig 2**shows the distributions of the value of filter function for the two groups, distinguishing ADHD patients from normal subjects. The values of the filter function are almost the same for with and without scrubbing the time points that showed large head motions (*i*.*e*., the framewise displacement > 1mm) when evaluating the functional connectivity (*r* = 0.9940 for the NYU and *r* = 0.9909 for the PU dataset). A one-way ANOVA found significant group differences in the values of the filter function (*p* < 0.0005, **Table 3**). We have not found any significant confounding effects of the phenotypic information, such as age, gender or medication status, to the group differences in the magnitude of the disease component (**Table C in S1 File**). Also, the value of the filter function, which measures the magnitude of the disease component, has excellent sensitivities and specificities (>96%) for the diagnosis of the children with ADHD at a cut-off score of 12 (11) for the NYU (PU) dataset (**Table D in S1 File**).

**Table 3:** Group means and standard deviations of the filter function value for TDC and three ADHD subtype groups.

Data Set	TDC	ADHD-C	ADHD-H	ADHD-I	*F*	*p*-value
NYU	9.2 (1.1)	14.4 (1.4)	17.3 (4.7)	14.7 (1.5)	289.4	<0.0005
PU	8.6 (1.1)	13.6 (1.6)		13.5 (1.7)	289.9	<0.0005

ADHD, attention-deficit/hyperactivity disorder; C, combined type; H, hyperactivity/impulsivity type; I, inattentive type; F, female; M, male; NYU, New York University Child Study Center; PU, Peking University; TDC, typically developing children

Group differences were evaluated using one-way analysis of variance

### Topological data analysis

**Fig 3:**
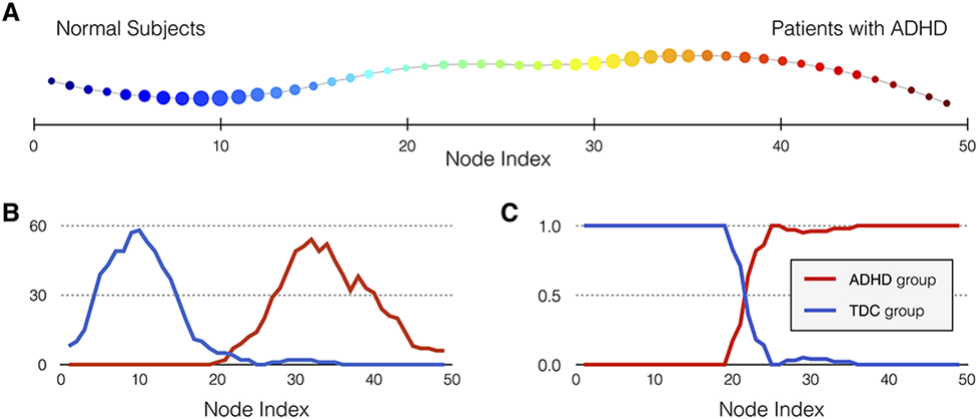
Resulting graph of the topological data analysis using the NYU dataset. (**A**) The ADHD-like subjects are ordered by the magnitude of deviation from the HSM. Each node is colored by the mean of the filter map on the points. Blue nodes contain normal-like subjects whose total deviations of the functional network from HSM are small (normal and normal-like subjects). Red nodes contain ADHD-like subjects whose deviations of the functional connectivity vector from HSM are large. The image of filter function was subdivided into 10 intervals with 85% overlap. (**B**) The number of subjects for each group versus node index. (**C**) The occupation ratio of group members in each node as function of node index.

Topological data analysis using *Mapper* was applied to the functional neuroimaging data and the chain-like graph was obtained as a result (**Fig 3**and **Table B in S1 File** for NYU data set). The blue-colored nodes contained mostly normal subjects, whereas red-colored nodes contained patients with ADHD who generally had large deviation from the functional network of the healthy subjects. The illustrations of the number of subjects and the occupation ratio of group members are presented in **Fig 3B and 3C**.

### Relationships between resulting topology and clinical phenotypic measures

**Fig 4:**
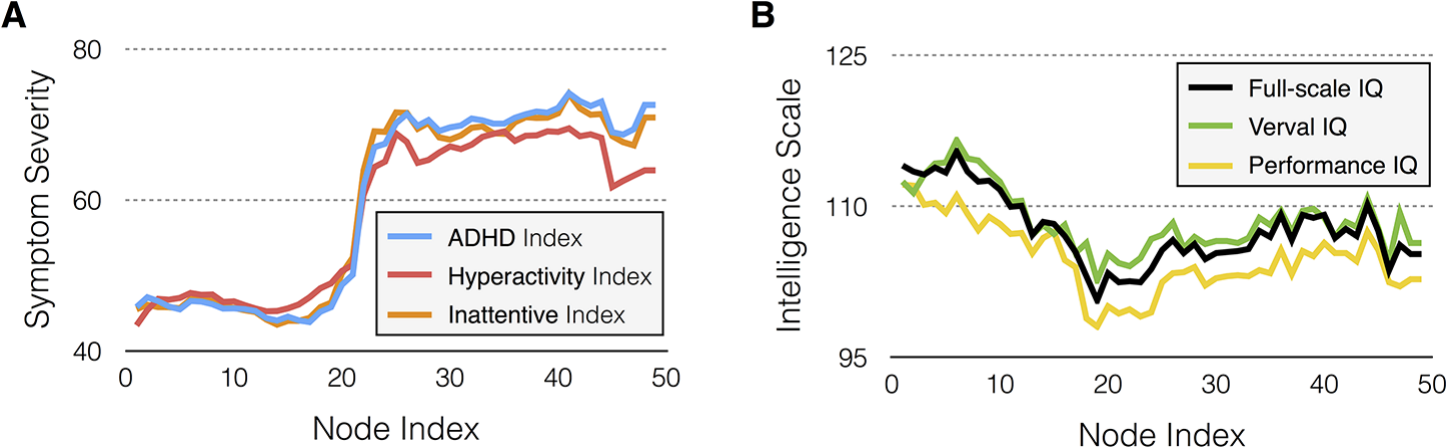
Visualization of the clinical phenotype data as a function of the node index in the NYU data. (**A**) Average symptom severity in each bin of graph. (**B**) Average intelligence scores in each bin of graph.

**Fig 5:**
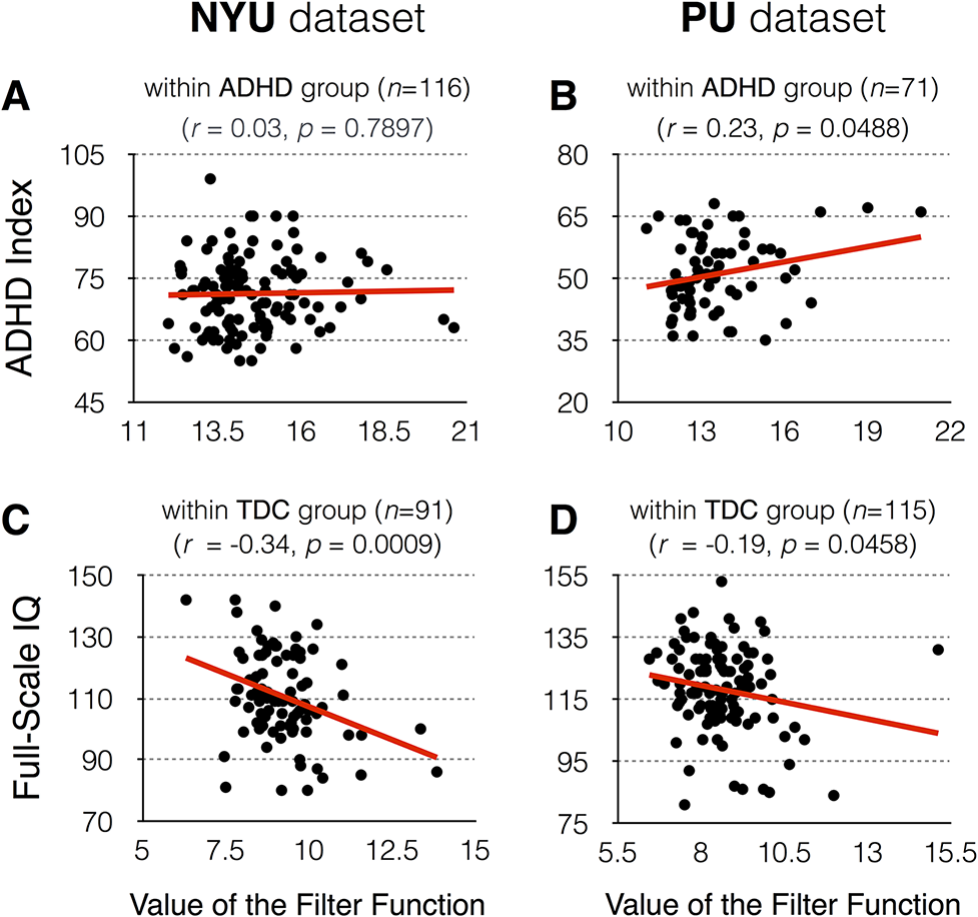
Scatter plots for the value of the filter function versus clinical phenotype variables. In the ADHD group, correlation coefficients with the ADHD index were evaluated for (**A**) the NYU dataset and (**B**) the PU dataset. In the TDC group, correlation coefficients with full-scale IQ were evaluated for (**C**) the NYU dataset and (**D**) PU dataset.

Since one important goal of topological data analysis is to obtain knowledge about the data followed by quantitative analysis, qualitative graphical (**Fig 4**) and quantitative correlation analyses (**Table 2**) were performed to find the hidden relationship between the *Mapper* results and clinical phenotypic variables. We inspected each node and computed the average value in each node. First, the visualization of the symptom severity as a function of node index revealed that the blue color nodes, whose index numbers ranged from 1 to 10, had significantly lower symptom severity than those of the red color nodes, whose index numbers ranged from 40 to 49 (**Fig 4A**). Significant statistical differences in the ADHD index, hyperactivity/impulsivity, and inattention scores between the blue and red color nodes were confirmed (*p* < 0.0005). Especially the symptom severity, represented by the ADHD index score from the PU dataset, showed positive correlations with the values of the filter function (*r* = 0.23, *p* = 0.0488; **Table 2**). However, for the NYU sample, the ADHD index did not show significant correlations with the value of the filter function. Second, the visualization of the intelligence scale as function of node index showed the decreasing trend across blue color nodes, which index number ranges from 1 to 19, and the constant trend across red color nodes, which index number ranges from 20 to 49 (**Fig 5B**). In the TDC group, we revealed that the significant negative correlations between the intelligence scales and the values of the filter function as described in **Table 2**. We compared 10 TDC subjects with the highest value of the filter function with the lowest values regarding demographic factors and ADHD symptom severity. The result shows that TDC subjects with higher values of the filter function consist of a younger age group (NYU dataset), contain more females (NYU dataset), have significantly lower IQ (NYU and PU datasets), and more severe hyperactivity/impulsivity (NYU dataset) compared to those with lower values of the filter function (**Table 4**). Finally, the correlation analyses found a significant positive relationship between the ratio of subjects with psychiatric comorbidity in each node and the node index for the NYU (*r* = 0.85, *p*<0.0005) and PU (*r* = 0.87, *p*<0.0005) datasets, respectively.

**Table 4:** Subgroup comparisons in demographics and clinical variables.

Demographic and clinical variables	New York University (NYU) dataset						Peking University (PU) dataset					
	TDC1 (low VFF)		TDC2 (high VFF)		*t*, *chi2*	*p*	TDC1 (low VFF)		TDC2 (high VFF)		*t*, *chi2*	*p*
	*n*	Mean (SD)	*n*	Mean (SD)			*n*	Mean (SD)	*n*	Mean (SD)		
Value of the filter function	10	7.6 (0.5)	10	1.2 (1.2)	-9.18	<0.0005	10	7.0 (0.3)	10	11.1 (1.5)	-8.08	<0.0005
Demographic Variables												
Gender (M/F)†		8/2		3/7	5.10	0.024	10	5/5	10	3/7	0.83	0.361
Age	10	13.8 (2.5)	10	10.7 (2.6)	2.72	0.014	10	11.3 (1.7)	10	11.9 (1.7)	-0.78	0.446
Symptom Severity												
ADHD Index	9	44.9 (5.6)	10	45.1 (4.6)	-0.09	0.929	7	26.1 (5.9)	10	29.0 (6.6)	-0.92	0.373
Inattentive	9	44.8 (6.0)	10	45.4 (4.2)	-0.27	0.794	7	14.0 (3.8)	10	16.2 (4.6)	-1.04	0.314
Hyper/Impulsive	9	43.3 (0.5)	10	47.8 (5.8)	-2.28	0.036	7	12.1 (2.9)	10	12.8 (3.4)	-0.42	0.684
Intelligence Quotient (IQ)												
Full-Scale IQ	10	117.7 (20.7)	10	97.7 (12.5)	2.61	0.018	10	123.0 (9.8)	10	105.2 (15.2)	3.11	0.006
Verbal IQ	10	115.9 (20.0)	10	101.7 (10.5)	1.99	0.062	10	120.4 (8.3)	10	107.2 (16.2)	2.30	0.034
Performance IQ	10	115.0 (18.1)	10	94.2 (13.8)	2.89	0.010	10	120.8 (12.1)	10	101.7 (16.1)	2.99	0.008

IQ, Intelligence quotient; SD, standard deviation; TDC1, 10 TDC subjects with the lowest values of the filter function; TDC2, 10 TDC subjects with the highest value of the filter function; VFF, value of the filter function.

†chi2 test were performed.

## Discussion

To the best of our knowledge, this is the first attempt to incorporate the HSM and topological data analysis for unveiling hidden relationships between clinical phenotypic variables of interest, such as IQ, symptom severity, and comorbidity rate of ADHD, and data on brain functional connectivity. The two methodologies were applied for the first time to create an objective measure of disease severity and a partial clustering model of patients with ADHD based on neuroimaging data. These two methods, when used in combination, might be promising tools to quantify disease components within brain networks.

### Interpretation of topological data analysis

The partial clustering methods through topological data analysis produced an easily recognizable chain-like graph (**Fig 3**). Originally, we expected that *Mapper* would yield a branch-shaped graph with two progressive arms; each arm differentiating the inattentive subtype of ADHD from the combined subtype, similar to two subtypes of breast cancer that were detected in a previous study [31]. However, unlike our hypothesis, our analysis could not identify discrete subgroups of ADHD to validate the DSM-IV subtype model of ADHD. This result is in line with previous studies questioning the discriminant validity of the DSM-IV ADHD subtypes. Studies of etiology, neuropsychological functioning, and treatment response do not provide enough evidence for the distinction between ADHD-I and ADHD-C subtypes, even though they provide support for the validity of the DSM-IV inattention and hyperactivity-impulsivity symptom dimensions [37]. In addition, the strongest argument against the ADHD subtype model is the instability of the subtype classification over time (only 35% meet criteria for the same subtype after 5 years) [38]. Recently, the nosology of ADHD subtypes has been updated in the DSM-V [39]: categorical subtypes of ADHD have been retained, but they are now referred to as combined presentation, predominantly inattentive presentation, and predominantly hyperactive/impulsive presentation. This wording change from “subtype” to “presentation” reflects the fluidity in how the symptoms of disorder may present in the same individual over time [38]. The model proposed by Lahey and Willcut (2010) defines ADHD as “a single disorder without subtypes, with dimensional modifiers that reflect the number of inattention and hyperactivity-impulsivity symptoms” [40]. Our results are consistent with this model of ADHD, in which the *Mapper* result is presented as a long gradual progression (**Fig 3**), showing ADHD symptoms as dimensional (or quantitative) traits that form a continuum with the normal state.

### Intelligence and the magnitude of disease component

According to our results (**Fig 5C and 5D**and **Table 2**), significant negative correlations between the magnitude of the disease component (represented by the filter value) and the IQ score were found in the TDC group but not in the ADHD group. Our results agree with recent studies showing that brain structure (e.g., cerebral gray matter volume and white matter microstructure) or small-world network parameters were associated with IQ for controls, but not for ADHD [41,42]. One possible explanation for these findings is that the relationship between clinical measures (e.g., IQ score) and brain functional measures may vary depending on the presence or absence of pathological processes. Therefore, certain relationships demonstrated in healthy individuals may not be observed in clinical populations. However, our result is rather unexpected, since it is well known that ADHD symptoms can interfere with performance in intelligence tests and adversely affect IQ scores [43]. In clinical practice, it is often observed that performance IQ in ADHD children is on average 7–10 points lower than that of comparisons [6]. A previous study also suggested a negative association between ADHD symptoms and IQ scores [43]. Taken together, further studies need to be done to clarify the relationship between IQ score and ADHD symptoms in this patient population.

In other applications, the identification of TDC subjects who present with sub-threshold ADHD symptoms is one of the key areas of biomarker research, because in mild forms of the disease it is difficult to distinguish between disorder and normal groups in clinical practice [21,44]. In these diagnostically ambiguous situations, *Mapper* presents its strength over standard clustering methods in that long gradual drifts in the graphs are visible, as shown in **Fig 3**, implying the continuity from normal to progressively advanced disease symptoms [31]. Thus, TDC subjects with higher values of the node index might be different from those with lower values, even though they are clustered within the same TDC group. Our analysis of TDC subjects with the highest value of the filter function showed that this subgroup differed significantly from the TDC subjects with the lowest value; they were of younger age, had significantly lower performance IQ, and more severe hyperactivity/impulsivity symptoms. A longitudinal follow up of this subgroup of TDC will be needed to investigate whether these individuals constitute a high-risk group who might eventually develop ADHD.

### Comorbidity, ADHD symptom severity and the magnitude of disease component

The presence of comorbidity has important implications for understanding assessment and treatment of patients with ADHD; thus, we examined the association between the ratio of subjects with comorbidity and the magnitude of disease component represented by the node index in each node. Although several studies reported that neuropsychological deficits are similar in patients with ADHD only and patients with ADHD plus comorbidity [45,46], other studies reported the opposite result proclaiming that patients with ADHD plus comorbidity have greater neuropsychological deficits than those with ADHD alone [47–49]. Our study supports the later view showing that the rates of comorbidity in ADHD increase when the disease component of functional connectivity (represented by the value of the filter function or node index) is large; the greater the node index is, the higher the rates of comorbidity in ADHD patients.

In addition, a significant positive correlation between the magnitude of the disease component represented by the value of the filter function and ADHD index score was found in the PU dataset (**Fig 5B**). However, significant correlations were not found in the NYU dataset (**Fig 5A**). Although the reasons for the different results from each institution are unclear at present, one possible explanation would be the difference in phenotypic characterization of the study samples at both sites. For example, ADHD symptoms were measured using different rating scales in each institution (PU used the ADHD Rating Scale IV; NYU used the Conners’ Parent Rating Scale-Revised, Long Version). Therefore, further studies to replicate the PU findings in different datasets are needed to reach a definitive conclusion.

### Limitations

The results mentioned above should be interpreted with caution with the following limitations in mind. First, the ADHD-200 data have site-specific differences in behavioral measurement, imaging data acquisition, scanner quality and protocols, and subject populations from the site contributing to the data. To overcome these problems, we analyzed the NYU and PU dataset separately, and mainly presented the results from the NYU site for this study (**Fig 2**). Nonetheless, the comparison of the NYU dataset with the independent PU dataset produced very similar results, except for the one result regarding the association between the value of the filter function and ADHD symptoms. Thus, the methodologies we propose may be considered as relatively unaffected by these sources of variation and bias caused by differences between study samples. Second, two groups were successfully identified on the basis of brain imaging data, such as the magnitude of the disease component, but phenotypic information such as gender, age, and medication status were not included in the topological data analysis. The demographic information may also contribute greatly to the disease classification, as shown in the competition results from the ADHD-200 dataset, which shows that diagnosis based on demographic variables outperforms imaging-based diagnostic prediction. Third, additional interaction effects of disease duration and medication may further impact the neurobiological substrate in specific ways [21] and future studies need to be consider these factors. Fourth, our findings may not demonstrate specificity to ADHD. Given our finding that subjects who are most different from controls (larger value of the filter function) have more comorbid disorders, the value of the filter function may reflect a brain of individuals who have more impairments. Fifth, The magnitude of disease component obtained from the healthy state model might not fully capture the complexity of the functional network, such as strength and direction of correlations among different brain regions. Yet, it could tell us the amount of deviation in the functional network as a single value, which is more convenient for further correlation analyses with clinical variables.

## Conclusions

Despite the limitations, we introduced the HSM and a topological clustering tool in the analysis of neuroimaging data for identifying a brain-phenotypic relationship. We found that the magnitude of the disease component obtained from HSM is significantly correlated with IQ scores in the TDC group, and the resulting topology contained the information of symptom severity or comorbidity rates of ADHD as function of node index. The application of HSM and topological data analysis methods to brain connectivity data might be a promising tool to quantify the disease component of ADHD and reveal the hidden relationship between clinical phenotypic variables and brain connectivity.

## Supporting Information

S1 FileSupplementary methods and results.Mean and standard deviations (SD) of the symptom severity and intelligence scale in NYU dataset and PU dataset **(Table A)**. **Number of subjects in each group (TDC and ADHD) for each bin of the output graph of *Mapper* for NYU dataset (Table B)**. The effects of the phenotypic information to the magnitude of the disease component **(Table C)**. The summary of receiver operating characteristic (ROC) analysis using the value of the filter function **(Table D)**. The decomposition of the original functional connectivity vector ***T***_***i***_ into the Normal component, which is the linear models fit $T_{i}^{\mathrm{Nc}}$ onto the Healthy State Model, and the disease component $T_{i}^{\mathrm{Dc}}$ vector of residuals. For example, decompositions of ***T***_***i***_ with (A) small and (B) large disease component were visualized (**Fig A**). The areas under the receiver operating characteristics (ROC) curves for the value of the filter function were illustrated for (A) the NYU and (B) PU dataset, respectively (**Fig B**). Schematic diagram of topological data analysis using *Mapper*. (A) The data is sampled from a noisy Y-shape point cloud in the two-dimensional space, and the filter function is *f(x*,*y) = y*. We divided the range of the filter into 5 intervals and a 50% overlap. (B) For each interval, we compute the clustering of the points lying within the domain of the filter restricted to the interval. Distributions of the distances from single linkage dendrogram in each filter bin. For example, distance distributions for 1^st^ and 9^th^ filter bin were presented. The summation of frequencies appeared after zero bins is the number of clusters, (C) Finally, we have the simplicial complex by connecting the clusters whenever they have non-empty intersection. The color of vertices represents the average filter value (**Fig C**). Sample application of *Mapper* to the Y-shape noisy point cloud. In this example illustration, 5 intervals with 20–80% overlaps and 10 intervals with 80% overlap are the appropriate choose of the input parameters of *Mapper* (**Fig D**). Sample application of *Mapper* to O-shape noisy point cloud data. In this example illustration, 5 intervals with 50–80% overlaps, 10 intervals with 50–80% overlaps, and 15 intervals with 80% overlap are the appropriate choose of the input parameters of *Mapper* (**Fig E**). Visualization of the clinical phenotype data as a function of the node index in the PU data: (A) The average symptom severity in each bin of graph; (B) The average intelligence scores in each bin of graph (**Fig F**).(DOCX)

## References

[1] Feldman HM, Reiff MI. Clinical practice. Attention deficit-hyperactivity disorder in children and adolescents. N Engl J Med. 2014;370: 838–846. 10.1056/NEJMcp1307215 24571756

[2] Biederman J, Faraone SV, Spencer TJ, Mick E, Monuteaux MC, Aleardi M. Functional impairments in adults with self-reports of diagnosed ADHD: A controlled study of 1001 adults in the community. J Clin Psychiatry. 2006;67: 524–540. 16669717 10.4088/jcp.v67n0403

[3] American Psychiatric Association. Diagnostic and Statistical Manual of Mental Disorders, Fourth Edition, Text Revision: American Psychiatric Association; 2000.

[4] Hyman SE. Neuroscience, genetics, and the future of psychiatric diagnosis. Psychopathology. 2002;35: 139–144. 12145499 10.1159/000065134

[5] Nesse RM, Stein DJ. Towards a genuinely medical model for psychiatric nosology. BMC Med. 2012;10: 5 10.1186/1741-7015-10-5 22244350 PMC3395863

[6] The ADHD-200 Consortium. The ADHD-200 Consortium: A Model to Advance the Translational Potential of Neuroimaging in Clinical Neuroscience. Front Syst Neurosci. 2012;6: 62 22973200 10.3389/fnsys.2012.00062 PMC3433679

[7] van Praag HM. Kraepelin, biological psychiatry, and beyond. Eur Arch Psychiatry Clin Neurosci. 2008;258 Suppl 2: 29–32. 10.1007/s00406-008-2006-1 18516514

[8] Getahun D, Jacobsen SJ, Fassett MJ, Chen W, Demissie K, Rhoads GG. Recent trends in childhood attention-deficit/hyperactivity disorder. JAMA Pediatr. 2013;167: 282–288. 10.1001/2013.jamapediatrics.401 23338799

[9] Weiler MD, Bellinger D, Simmons E, Rappaport L, Urion DK, Mitchell W, et al Reliability and Validity of a DSM-IV Based ADHD Screener. Child Neuropsychology. 2000;6: 3–23. 10980665 10.1076/0929-7049(200003)6:1;1-B;FT003

[10] Castellanos FX, Proal E. Large-scale brain systems in ADHD: beyond the prefrontal-striatal model. Trends Cogn Sci. 2012;16: 17–26. 10.1016/j.tics.2011.11.007 22169776 PMC3272832

[11] Faraone SV, Biederman J. Neurobiology of attention-deficit hyperactivity disorder. Biol Psychiatry. 1998;44: 951–958. 9821559 10.1016/s0006-3223(98)00240-6

[12] Konrad K, Eickhoff SB. Is the ADHD brain wired differently? A review on structural and functional connectivity in attention deficit hyperactivity disorder. Human Brain Mapping. 2010;31: 904–916. 10.1002/hbm.21058 20496381 PMC6871159

[13] Shaw P, Eckstrand K, Sharp W, Blumenthal J, Lerch JP, Greenstein D, et al Attention-deficit/hyperactivity disorder is characterized by a delay in cortical maturation. Proc Natl Acad Sci U S A. 2007;104: 19649–19654. 18024590 10.1073/pnas.0707741104 PMC2148343

[14] Tripp G, Wickens JR. Neurobiology of ADHD. Neuropharmacology. 2009;57: 579–589. 10.1016/j.neuropharm.2009.07.026 19627998

[15] Bullmore E. The future of functional MRI in clinical medicine. Neuroimage. 2012;62: 1267–1271. 10.1016/j.neuroimage.2012.01.026 22261374

[16] Bullmore E, Fletcher P, Jones PB. Why psychiatry can’t afford to be neurophobic. The British Journal of Psychiatry. 2009;194: 293–295. 10.1192/bjp.bp.108.058479 19336776

[17] Craddock RC, Holtzheimer PE 3rd, Hu XP, Mayberg HS. Disease state prediction from resting state functional connectivity. Magn Reson Med. 2009;62: 1619–1628. 10.1002/mrm.22159 19859933 PMC3749911

[18] Dosenbach NU, Nardos B, Cohen AL, Fair DA, Power JD, Church JA, et al Prediction of individual brain maturity using fMRI. Science. 2010;329: 1358–1361. 10.1126/science.1194144 20829489 PMC3135376

[19] Ecker C, Marquand A, Mourao-Miranda J, Johnston P, Daly EM, Brammer MJ, et al Describing the brain in autism in five dimensions—magnetic resonance imaging-assisted diagnosis of autism spectrum disorder using a multiparameter classification approach. J Neurosci. 2010;30: 10612–10623. 10.1523/JNEUROSCI.5413-09.2010 20702694 PMC6634684

[20] Fox MD, Greicius M. Clinical applications of resting state functional connectivity. Front Syst Neurosci. 2010;4: 19 10.3389/fnsys.2010.00019 20592951 PMC2893721

[21] Kloppel S, Abdulkadir A, Jack CR Jr., Koutsouleris N, Mourao-Miranda J, Vemuri P. Diagnostic neuroimaging across diseases. Neuroimage. 2012;61: 457–463. 10.1016/j.neuroimage.2011.11.002 22094642 PMC3420067

[22] Michel CM, Murray MM. Towards the utilization of EEG as a brain imaging tool. NeuroImage. 2012;61: 371–385. 10.1016/j.neuroimage.2011.12.039 22227136

[23] Seidman LJ, Valera EM, Makris N, Monuteaux MC, Boriel DL, Kelkar K, et al Dorsolateral prefrontal and anterior cingulate cortex volumetric abnormalities in adults with attention-deficit/hyperactivity disorder identified by magnetic resonance imaging. Biol Psychiatry. 2006;60: 1071–1080. 16876137 10.1016/j.biopsych.2006.04.031

[24] Zhu C-Z, Zang Y-F, Cao Q-J, Yan C-G, He Y, Jiang T-Z, et al Fisher discriminative analysis of resting-state brain function for attention-deficit/hyperactivity disorder. NeuroImage. 2008;40: 110–120. 10.1016/j.neuroimage.2007.11.029 18191584

[25] Brown MR, Sidhu GS, Greiner R, Asgarian N, Bastani M, Silverstone PH, et al ADHD-200 Global Competition: diagnosing ADHD using personal characteristic data can outperform resting state fMRI measurements. Front Syst Neurosci. 2012;6: 69 10.3389/fnsys.2012.00069 23060754 PMC3460316

[26] Dey S, Rao AR, Shah M. Exploiting the brain's network structure in identifying ADHD subjects. Front Syst Neurosci. 2012;6: 75 10.3389/fnsys.2012.00075 23162440 PMC3499771

[27] Eloyan A, Muschelli J, Nebel MB, Liu H, Han F, Zhao T, et al Automated diagnoses of attention deficit hyperactive disorder using magnetic resonance imaging. Front Syst Neurosci. 2012;6: 61 10.3389/fnsys.2012.00061 22969709 PMC3431009

[28] Hyman SE. Can neuroscience be integrated into the DSM-V? Nat Rev Neurosci. 2007;8: 725–732. 17704814 10.1038/nrn2218

[29] Nestler EJ, Hyman SE. Animal models of neuropsychiatric disorders. Nat Neurosci. 2010;13: 1161–1169. 10.1038/nn.2647 20877280 PMC3750731

[30] Lum PY, Singh G, Lehman A, Ishkanov T, Vejdemo-Johansson M, Alagappan M, et al Extracting insights from the shape of complex data using topology. Sci. Rep. 2013;3. 10.1038/srep01236 PMC3566620 23393618

[31] Nicolau M, Levine AJ, Carlsson G. Topology based data analysis identifies a subgroup of breast cancers with a unique mutational profile and excellent survival. Proc Natl Acad Sci U S A. 2011;108: 7265–7270. 10.1073/pnas.1102826108 21482760 PMC3084136

[32] Nicolau M, Tibshirani R, Borresen-Dale AL, Jeffrey SS. Disease-specific genomic analysis: identifying the signature of pathologic biology. Bioinformatics. 2007;23: 957–965. 17277331 10.1093/bioinformatics/btm033

[33] Yao Y, Sun J, Huang X, Bowman GR, Singh G, Lesnick M, et al Topological methods for exploring low-density states in biomolecular folding pathways. J Chem Phys. 2009;130: 144115 10.1063/1.3103496 19368437 PMC2719471

[34] Singh G, Memoli F, Carlsson G. Topological Methods for the Analysis of High Dimensional Data Sets and 3D Object Recognition. Eurographics Symposium on Point-Based Graphics. 2007: 1–11.

[35] Tomasi D, Volkow ND. Abnormal functional connectivity in children with attention-deficit/hyperactivity disorder. Biol Psychiatry. 2012;71: 443–450. 10.1016/j.biopsych.2011.11.003 22153589 PMC3479644

[36] Carlsson G. Topology and data. Bull. Amer. Math. Soc. 2009;46: 255–308.

[37] Woo BS, Rey JM. The validity of the DSM-IV subtypes of attention-deficit/hyperactivity disorder. Aust N Z J Psychiatry. 2005;39: 344–353. 15860021 10.1080/j.1440-1614.2005.01580.x

[38] Willcutt EG, Nigg JT, Pennington BF, Solanto MV, Rohde LA, Tannock R, et al Validity of DSM-IV attention deficit/hyperactivity disorder symptom dimensions and subtypes. J Abnorm Psychol. 2012;121: 991–1010. 10.1037/a0027347 22612200 PMC3622557

[39] American Psychiatric Association. Diagnostic and Statistical Manual of Mental Disorders, 5th Edition: American Psychiatric Association; 2013.

[40] Lahey BB, Willcutt EG. Predictive validity of a continuous alternative to nominal subtypes of attention-deficit/hyperactivity disorder for DSM-V. J Clin Child Adolesc Psychol. 2010;39: 761–775. 10.1080/15374416.2010.517173 21058124 PMC3056555

[41] de Zeeuw P, Schnack HG, van Belle J, Weusten J, van Dijk S, Langen M, et al Differential brain development with low and high IQ in attention-deficit/hyperactivity disorder. PLoS One. 2012;7: e35770 10.1371/journal.pone.0035770 22536435 PMC3335015

[42] Wang L, Zhu C, He Y, Zang Y, Cao Q, Zhang H, et al Altered small-world brain functional networks in children with attention-deficit/hyperactivity disorder. Hum Brain Mapp. 2009;30: 638–649. 10.1002/hbm.20530 18219621 PMC6870909

[43] Kuntsi J, Eley TC, Taylor A, Hughes C, Asherson P, Caspi A, et al Co-occurrence of ADHD and low IQ has genetic origins. American Journal of Medical Genetics Part B: Neuropsychiatric Genetics. 2004;124B: 41–47. 10.1002/ajmg.b.20076 14681911

[44] Balazs J, Kereszteny A. Subthreshold attention deficit hyperactivity in children and adolescents: a systematic review. Eur Child Adolesc Psychiatry. 2014;23: 393–408. 10.1007/s00787-013-0514-7 24399038

[45] Klorman R, Hazel-Fernandez LA, Shaywitz SE, Fletcher JM, Marchione KE, Holahan JM, et al Executive Functioning Deficits in Attention‐Deficit/Hyperactivity Disorder Are Independent of Oppositional Defiant or Reading Disorder. Journal of the American Academy of Child & Adolescent Psychiatry. 1999;38: 1148–1155. 10504814 10.1097/00004583-199909000-00020

[46] Seidman LJ, Biederman J, Faraone SV, Milberger S, Norman D, Seiverd K, et al Effects of Family History and Comorbidity on the Neuropsychological Performance of Children with ADHD: Preliminary Findings. Journal of the American Academy of Child & Adolescent Psychiatry. 1995;34: 1015–1024. 7665440 10.1097/00004583-199508000-00011

[47] Johnson BD, Altmaier EM, Richman LC. Attention deficits and reading disabilities: Are immediate memory defects additive? Developmental Neuropsychology. 1999;15: 213–226.

[48] Korkman M, Pesonen AE. A comparison of neuropsychological test profiles of children with attention deficit-hyperactivity disorder and/or learning disorder. J Learn Disabil. 1994;27: 383–392. 8051511 10.1177/002221949402700605

[49] Mayes SD, Calhoun SL, Crowell EW. Learning Disabilities and ADHD: Overlapping Spectrum Disorders. Journal of Learning Disabilities. 2000;33: 417–424. 15495544 10.1177/002221940003300502

