# Supplementary material for: A New Approach to Investigate the Association between Brain Functional Connectivity and Disease Characteristics of Attention-Deficit/Hyperactivity Disorder: Topological Neuroimaging Data Analysis
Source: PLoS One. 2015 Sep 9;10(9):e0137296. doi: 10.1371/journal.pone.0137296 (PMC4564101; doi:10.1371/journal.pone.0137296)
Supplement: S1 File — Mean and standard deviations (SD) of the symptom severity and intelligence scale in NYU dataset and PU dataset (Table A). Number of subjects in each group (TDC and ADHD) for each bin of the output graph of Mapper for NYU dataset (Table B). The effects of the phenotypic information to the magnitude of the disease component (Table C). The summary of receiver operating characteristic (ROC) analysis using the value of the filter function (Table D). The decomposition of the original functional connectivity vector T i into the Normal component, which is the linear models fit TiNc onto the Healthy State Model, and the disease component TiDc vector of residuals. For example, decompositions of T i with (A) small and (B) large disease component were visualized (Fig A). The areas under the receiver operating characteristics (ROC) curves for the value of the filter function were illustrated for (A) the NYU and (B) PU dataset, respectively (Fig B). Schematic diagram of topological data analysis using Mapper. (A) The data is sampled from a noisy Y-shape point cloud in the two-dimensional space, and the filter function is f(x,y) = y. We divided the range of the filter into 5 intervals and a 50% overlap. (B) For each interval, we compute the clustering of the points lying within the domain of the filter restricted to the interval. Distributions of the distances from single linkage dendrogram in each filter bin. For example, distance distributions for 1st and 9th filter bin were presented. The summation of frequencies appeared after zero bins is the number of clusters, (C) Finally, we have the simplicial complex by connecting the clusters whenever they have non-empty intersection. The color of vertices represents the average filter value (Fig C). Sample application of Mapper to the Y-shape noisy point cloud. In this example illustration, 5 intervals with 20–80% overlaps and 10 intervals with 80% overlap are the appropriate choose of the input parameters of Mapper (Fig D). Sample app [file pone.0137296.s001.docx]

# A New Approach to Investigate the Association between Brain Functional Connectivity and Disease Characteristics of Attention-Deficit/Hyperactivity Disorder: Topological Neuroimaging Data Analysis

Sunghyon Kyeong Seonjeong Park Keun-Ah Cheon Jae-Jin Kim Dong-Ho Song Eunjoo Kim^*^

*Supplementary Information*

#### Functional Image Preprocessing (“Athena Pipeline”)

#### The current study used the preprocessed resting state functional network data which can be obtained from Athena website (<http://www.nitrc.org/plugins/mwiki/index.php/neurobureau:AthenaPipeline>). The dataset were preprocessed according to the Athena pipeline as following: exclusion of the first four echo-planar image (EPI) volume; slice timing correction; deoblique dataset; realignment for head motion correction (first volume as reference); masking the volumes to exclude voxels at non-brain regions; averaging the EPI volumes to obtain a mean functional image; co-registration of this mean image to the respective anatomic image of the subject; spatial transformation of functional data into template space (4x4x4 mm^3^); extraction of resting state fMRI time courses from white matter (WM) and cerebrospinal-fluid (CSF) using masks obtained from segmenting the structural data; removing effects of WM, CSF, head motion, and trend using linear multiple regression and holding the residuals; temporal band-pass filter (0.009 < f < 0.08 Hz); spatial smoothing the filtered data using a Gaussian filter (full-width at half maximum = 6mm).

#### After performing the spatio-temporal preprocessing, functional connectivity was measured as the correlation coefficients of time courses of any two regions of interest (ROIs). In this study, we used the automated anatomical labeling (AAL) parcellation [1] to extract time courses of 116 ROIs (ADHD200_AAL_TCs_filtfix.tar.gz).

#### Healthy State Model

The functional connectivity vector $T_{i}$ of each subject can be decomposed into the normal component and the disease component by disease-specific connectome analysis, which is a connectome version of disease-specific genomic analysis [2] as follows:

|  | $T_{i}=T_{i}^{Nc}+T_{i}^{Dc},$ | Eq. (1) |
| --- | --- | --- |

where the normal component (Nc) of data mimics healthy state model (HSM).

Figure A. The decomposition of the original functional connectivity vector $\boldsymbol{T}_{\boldsymbol{i}}$ into the Normal component, which is the linear models fit $\boldsymbol{T}_{\boldsymbol{i}}^{\boldsymbol{Nc}}$ onto the Healthy State Model, and the disease component $\boldsymbol{T}_{\boldsymbol{i}}^{\boldsymbol{Dc}}$ vector of residuals. For example, decompositions of $\boldsymbol{T}_{\boldsymbol{i}}$ with (a) small and (b) large disease component were visualized.

The healthy state model was to describe the deviation from the normal state (Figure A). The normal component of each subject can be estimated by the HSM. In this study, the HSM was obtained from the dataset in the TDC group rather than whole dataset. The *FLAT* construction (Nicolau et al., 2007), which is a combination of mathematical data reconstruction followed by dimension reduction through principal component analysis (PCA), was used to form the HSM. *FLAT* construction is a method to de-sparse the high dimensional data by substituting for each normal subject data $T_{i}$ and its fit $\hat{T}_{i}$ to a linear model in the other normal subject data:

|  | $\hat{T}_{i}=\sum_{k\in N,k\neq i} \beta_{k}T_{k}+\epsilon.$ | Eq. (2) |
| --- | --- | --- |

Roughly, working with *FLAT* vectors $\hat{T}_{i}$ is intended to reduce aspects of the data that are unique to each normal subject vector $T_{i}$ and it is more robust. Also, the *FLAT* constructions give more neurobiologically meaningful information that is unique to $T_{i}$ (Nicolau et al., 2007). Thus, we applied the PCA to dimension reduction using *FLAT* vectors as inputs.

|  | $\hat{D}^{'}=F\times\boldsymbol{H},$ | Eq. (3) |
| --- | --- | --- |

where $F$ is the *n*-by-*p* reducing matrix (number of normal subjects by number of principal components) and $\boldsymbol{H}$ is the *p*-by-*m* matrix that represents normal spaces (components by number of network edges). Each column of $F$ represents the weight vector for each row of $\boldsymbol{H}$. We chose 55 principal components from 98 normal subjects using the *FLAT* construction. The use of 55 components sufficiently explained over 99% variance of whole normal subjects’ functional network data $\hat{D}'$.

The PCA on a graph was proposed to reveal hidden patterns in networks. For example, the researchers identified functional connectivity patterns, so-called “eigenconnectivities”, which reflect meaningful neurobiological connectivity patterns in resting state fMRI data [3, 4]. In the methodological point of view, decomposing of the functional network data by $\boldsymbol{H}$ is equivalent to reconstruct the functional connectivity using eigenconnectivities that are identified from normal subjects. However, FLAT construction generates more robust results.

The normal component of $T_{i}$ can be described by linear fitting to $\boldsymbol{H}$ as follows:

|  | $T_{i}=\sum_{k=1}^{55} \beta_{k}\boldsymbol{H}_{k}+\epsilon,$ | Eq. (4) |
| --- | --- | --- |

where $\boldsymbol{H}_{k}$ represents the *k*-th row of $\boldsymbol{H}$, i.e., the *k*-th normal space or principal component. Once the fitting parameters ($\beta$’s) are determined by Eq. (4), we can compute the normal component of $T_{i}$ in Eq. (1) as follows:

|  | $T_{i}^{Nc}=\sum_{k=1}^{55} \beta_{k}\boldsymbol{H}_{k}.$ | Eq. (5) |
| --- | --- | --- |

**Relationships between the magnitude of the disease components and the phenotypic information**

To check the effects of phenotypic variables in the magnitude of the disease components, we performed the general linear model with the magnitude of the disease component as the dependent variable and the group indicator as the independent variable. In addition, we included the age, gender, or medication status as a covariate in the separate model as following:

|  | $Y=\beta_{0}+\beta_{1}X+ \beta_{2}Z+\varepsilon,$ | Eq. (6) |
| --- | --- | --- |

where *Y* is the magnitude of the disease component, *X* is a group indicator, and *Z* is the age, gender, or medication status. According to the general linear modeling, the phenotypic information such the age, gender, and medication status are not significantly related to the magnitude of the disease component (Table C). Here we note that the medication status in the NYU dataset was not fully available in the children with ADHD.

#### Receiver operating characteristic analysis

We quantitatively assessed an area under the receiver operating characteristics (ROC) curve (Figure B) including estimation of sensitivity and specificity. This analysis was performed to evaluate the possibility of a new biomarker, *i.e.*, the value of the filter function, for the clinical purpose. We computed the sensitivities and specificities for each dataset and summarized in Table D. Also, we could qualitatively check the classification performance by looking at the distribution of the value of the filter function. Figure 2 of the main manuscript showed clear differences of the value of the filter function between typically developing controls and the children with ADHD.


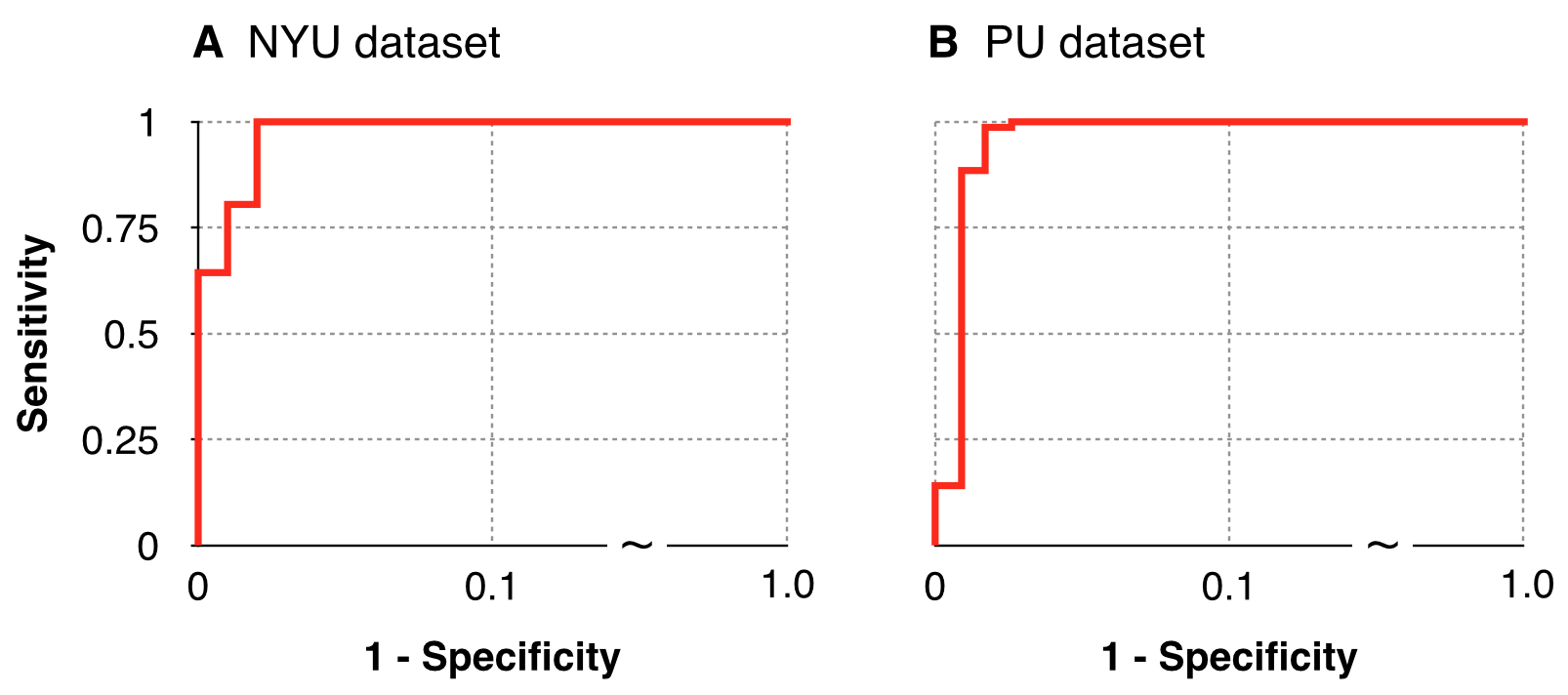


Figure B. The areas under the receiver operating characteristics (ROC) curves for the value of the filter function were illustrated for (A) the NYU and (B) PU dataset, respectively.

#### Topological Data Analysis

The topological data analysis (TDA) is to characterize the topology of high dimensional shapes of point clouds. The method is effective in dealing with a high degree of heterogeneity in distribution, capturing structural features such as flares in the dataset. In particular, a technique called Mapper is used to produce topological models induced by point clouds. As illustrated in Figure C, the points are divided using filter functions and then clustered into closely connected groups. Whenever groups overlap, they are connected, producing a simplified topological model for the space suggested by the data points. The role of the filter function is to collapse the high dimensional data into a single point. The L2-norm, L-infinity norm, density function, eccentricity, eigenvalue of the first principal component are widely used examples of the filter function that carry interesting geometric information about datasets. Then, the values of the filter function were divided based on the parameters such as interval and overlap. In general, increasing the number of intervals will increase the number of clusters we observe and decreasing the number of intervals will reduce it. Now, the distance matrix can be obtained by computing a distance of all pairwise points. The distance function has not necessarily to be a Euclidean distance. The essence of TDA is the *partial clustering*. For each bin of the filter, partial clustering and building a simplicial complex were performed. For example, as described in Figure B, if **F1** (dataset in 1^st^ filter bin) and **F2** (dataset in 2^nd^ filter bin) are subsets of the dataset, and $\mathbf{F1}\cap\mathbf{F2}$ is non-empty, then the clusters obtained from **F1** and **F2** may have non-empty intersections, and these intersections are used in building a topology of the dataset, which we called as a simplicial complex.


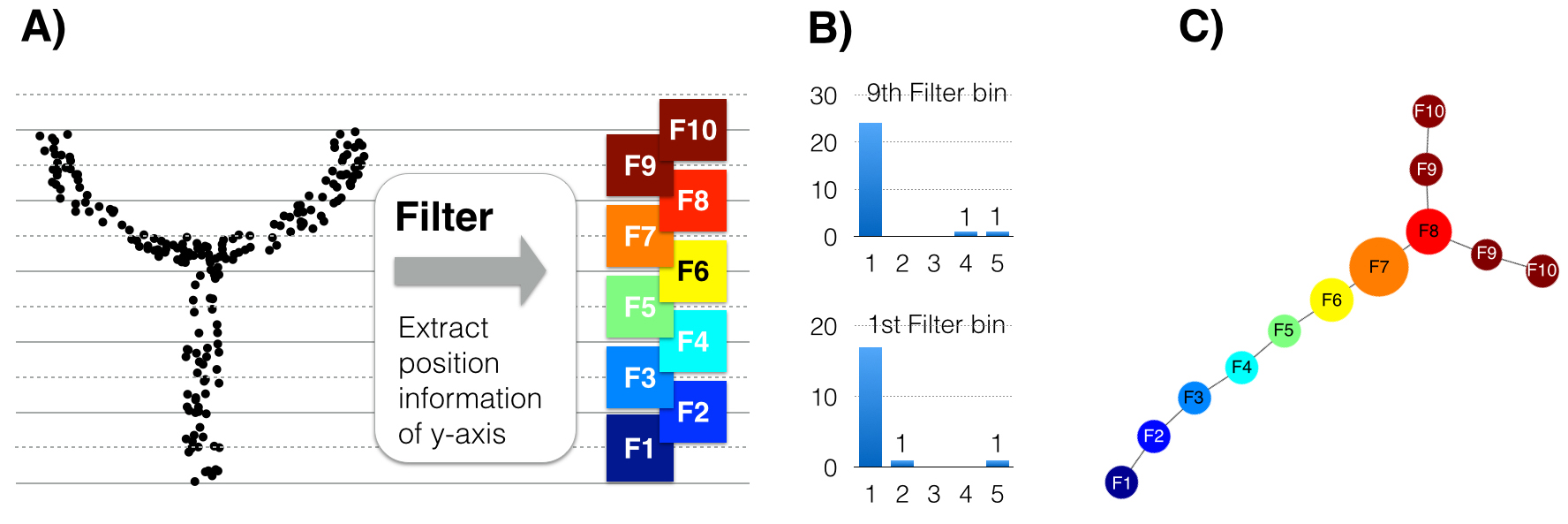


Figure C. Schematic diagram of topological data analysis using *Mapper*. (A) The data is sampled from a noisy Y-shape point cloud in the two-dimensional space, and the filter function is *f(x,y) = y*. We divided the range of the filter into 5 intervals and a 50% overlap. (B) For each interval, we compute the clustering of the points lying within the domain of the filter restricted to the interval. Distributions of the distances from single linkage dendrogram in each filter bin. For example, distance distributions for 1^st^ and 9^th^ filter bin were presented. The summation of frequencies appeared after zero bins is the number of clusters, (C) Finally, we have the simplicial complex by connecting the clusters whenever they have non-empty intersection. The color of vertices represents the average filter value.

**Applications of TDA to simulated sample data**

In this section, we illustrated two examples of the Mapper algorithm. Our aim is to demonstrate the usefulness of reducing a point cloud to a much smaller simplicial complex in the synthetic examples. We generated Y-shape noisy point cloud that has two flares. Also, we generated O-shape noisy point cloud. Using filter function f(x,y)=y, we visualized the topology of Y- and O-shape noisy point cloud with various combination of interval and overlap parameters. TDA captured the topology of Y-shape noisy data for the 5 intervals with 20-80% overlap and also 10 intervals with 80% overlap (Figure D). Similarly, TDA captured the topology of O-shape noisy data for the 5 intervals with 50-80% overlap, 10 intervals with 50-80% overlap, and 15 intervals with 80% overlap (Figure E).


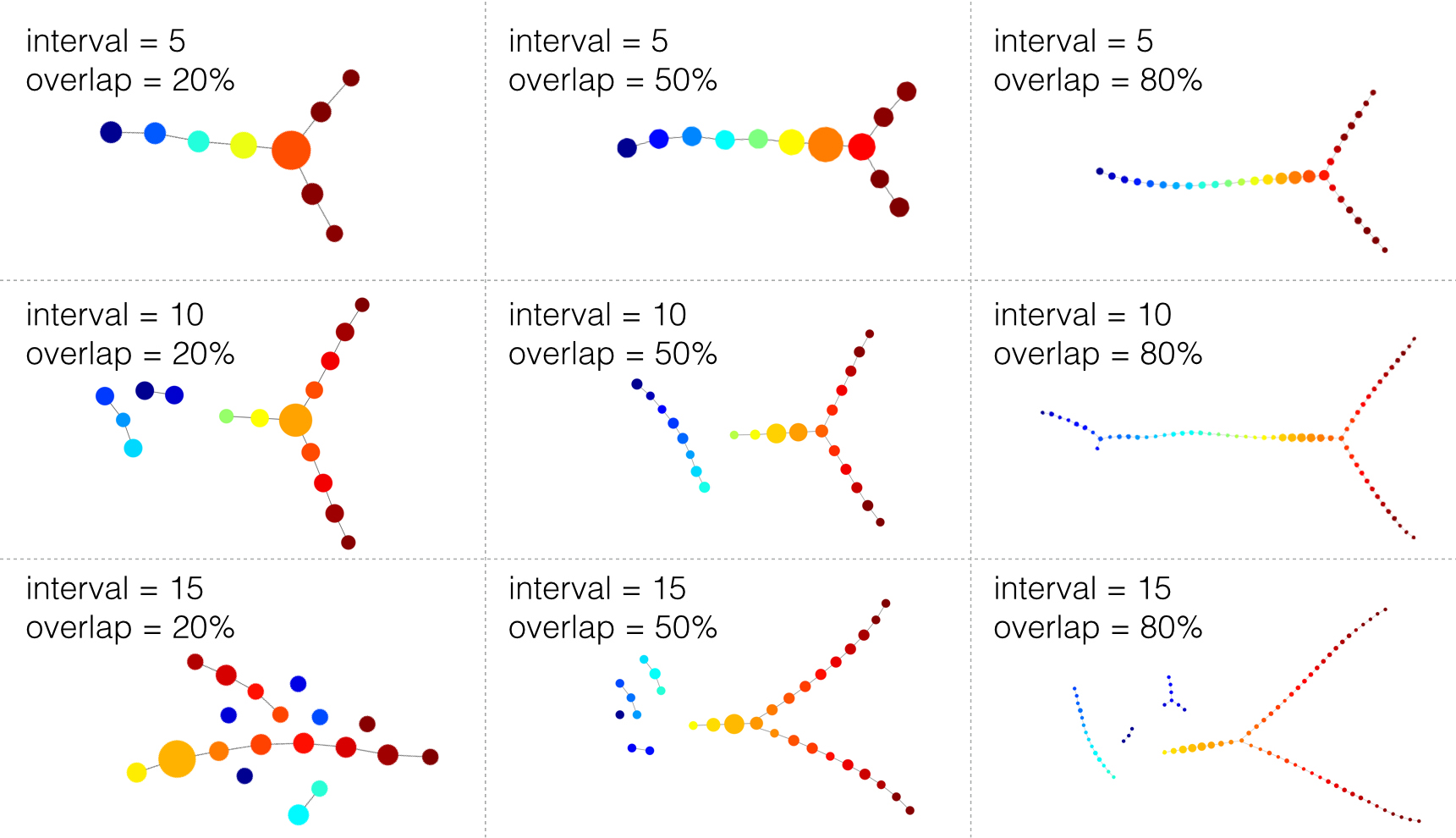


Figure D. Sample application of *Mapper* to the Y-shape noisy point cloud. In this example illustration, 5 intervals with 20-80% overlaps and 10 intervals with 80% overlap are the appropriate choose of the input parameters of *Mapper*.


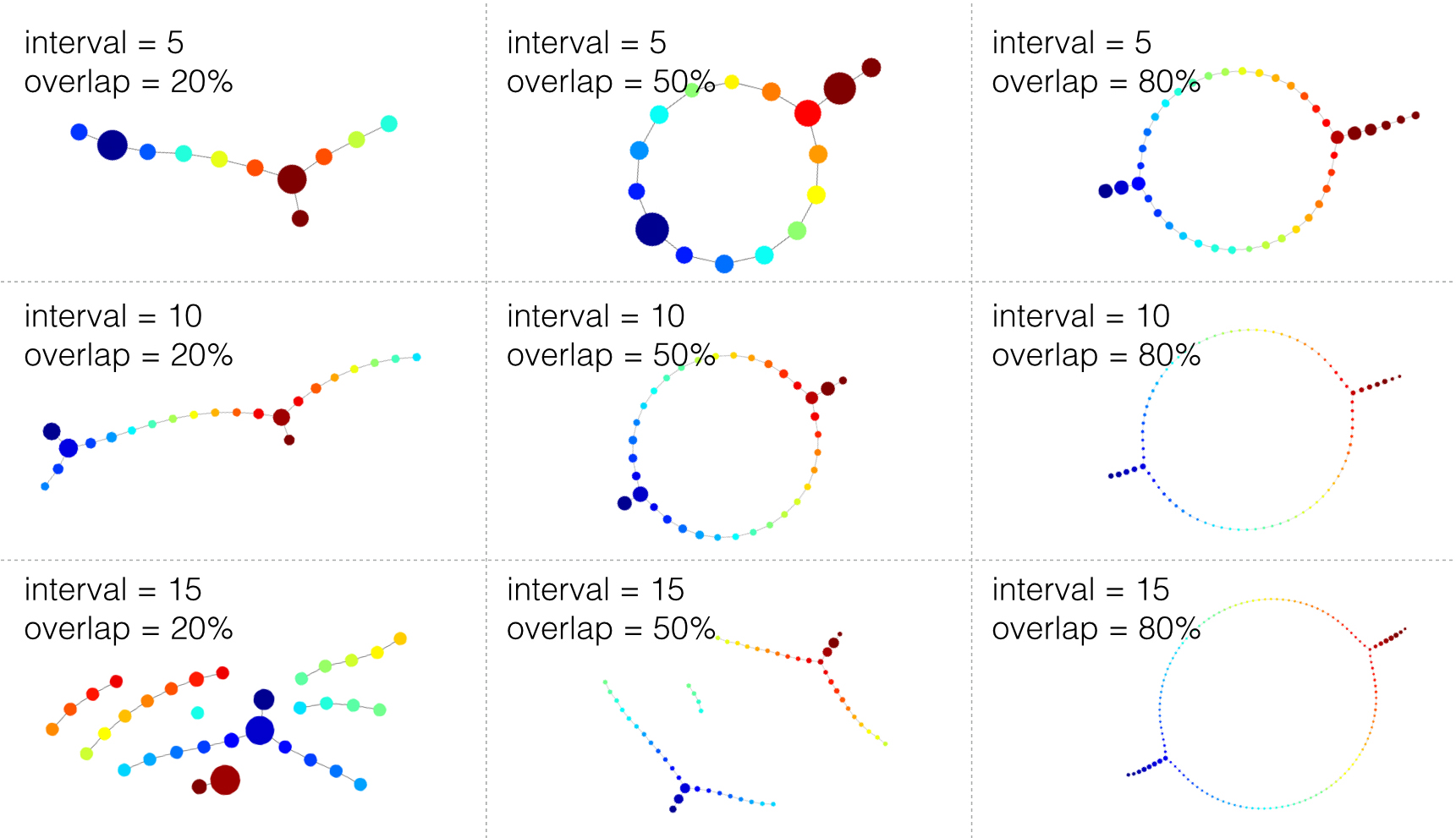


Figure E. Sample application of *Mapper* to O-shape noisy point cloud data. In this example illustration, 5 intervals with 50-80% overlaps, 10 intervals with 50-80% overlaps, and 15 intervals with 80% overlap are the appropriate choose of the input parameters of *Mapper*.

**Replication Results using PU data**

As we shown in NYU dataset, the relationships between resulting topology and clinical measures in PU dataset were investigated. The quantitative graphical illustration (Figure F) was performed to find the hidden relationship between the *Mapper* results and clinical measures.


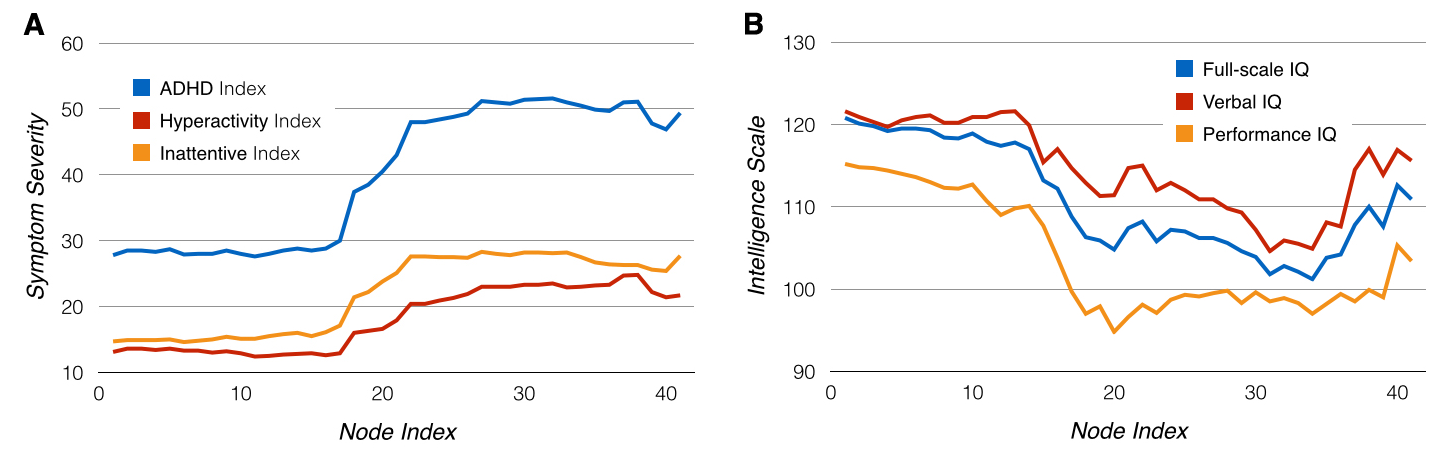


Figure F. Visualization of the clinical phenotype data as a function of the node index in the PU data. (A) The average symptom severity in each bin of graph. (B) The average intelligence scores in each bin of graph.

**References**

1. Tzourio-Mazoyer N, Landeau B, Papathanassiou D, Crivello F, Etard O, Delcroix N, et al. Automated anatomical labeling of activations in SPM using a macroscopic anatomical parcellation of the MNI MRI single-subject brain. NeuroImage. 2002;15(1):273-89. doi: 10.1006/nimg.2001.0978. PubMed PMID: 11771995.

2. Nicolau M, Tibshirani R, Borresen-Dale AL, Jeffrey SS. Disease-specific genomic analysis: identifying the signature of pathologic biology. Bioinformatics. 2007;23(8):957-65. doi: 10.1093/bioinformatics/btm033. PubMed PMID: 17277331.

3. Leonardi N, Richiardi J, Gschwind M, Simioni S, Annoni JM, Schluep M, et al. Principal components of functional connectivity: a new approach to study dynamic brain connectivity during rest. NeuroImage. 2013;83:937-50. doi: 10.1016/j.neuroimage.2013.07.019. PubMed PMID: 23872496.

4. Park B, Kim DS, Park HJ. Graph independent component analysis reveals repertoires of intrinsic network components in the human brain. PloS one. 2014;9(1):e82873. doi: 10.1371/journal.pone.0082873. PubMed PMID: 24409279; PubMed Central PMCID: PMC3883640.

Table A. Mean and standard deviations (SD) of the symptom severity and intelligence scale in NYU dataset and PU dataset

| Clinical phenotype | NYU dataset | | PU dataset | | *t* | *p*-value |
| --- | --- | --- | --- | --- | --- | --- |
|  | *n* | Mean (SD) | *n* | Mean (SD) |  |  |
| Symptom Severity |  |  |  |  |  |  |
| ADHD Score | 212 | 59.5 (15.0) | 172 | 37.6 (13.5) | 14.85 | <0.0005 |
| Inattentive Score | 212 | 59.0 (14.8) | 172 | 20.5 (7.5) | 31.09 | <0.0005 |
| Hyperactivity/Impulsivity | 212 | 58.2 (14.5) | 172 | 17.1 (6.9) | 34.25 | <0.0005 |
| Intelligence Scale |  |  |  |  |  |  |
| Full-Scale IQ | 206 | 108.3 (14.3) | 193 | 113.0 (14.7) | -3.25 | 0.001 |
| Performance IQ | 206 | 105.4 (14.6) | 193 | 106.7 (15.7) | -0.83 | 0.405 |
| Verbal IQ | 206 | 109.1 (14.1) | 193 | 116.0 (15.1) | -4.73 | <0.0005 |

The different number of data (*n*) is because of missing information.

**Table B.** Number of subjects in each group (TDC and ADHD) for each bin of the output graph of *Mapper* for NYU dataset

|  | 1 | 2 | 3 | 4 | 5 |
| --- | --- | --- | --- | --- | --- |
| Node index (1–5) | 8/0 | 10/0 | 15/0 | 27/0 | 39/0 |
| Node index (6–10) | 43/0 | 49/0 | 49/0 | 57/0 | 58/0 |
| Node index (11–15) | 53/0 | 49/0 | 42/0 | 37/0 | 28/0 |
| Node index (16–20) | 19/0 | 11/0 | 10/0 | 7/0 | 5/1 |
| Node index (21–25) | 5/2 | (4/7)*^a^* | 2/9 | 2/12 | 0/14 |
| Node index (26–30) | 0/20 | 1/29 | 1/33 | 2/41 | 2/49 |
| Node index (31–35) | 2/51 | 2/54 | 1/49 | 1/52 | 1/45 |
| Node index (36–40) | 0/39 | 0/32 | 0/38 | 0/33 | 0/31 |
| Node index (41–45) | 0/24 | 0/21 | 0/20 | 0/14 | 0/8 |
| Node index (46–49) | 0/7 | 0/7 | 0/6 | 0/6 |  |

*^a^*For example, the numbers in the parentheses represent the number of subjects in two groups in the 22nd bin of the graph: 4 for the TDC group and 7 for the ADHD group. The number of normal subjects is 98 and the number of patients with ADHD is 118.

This table is the quantitative description for Figure 1. The value of the filter function was subdivided into 10 intervals with 85% overlap. Therefore, the first node index is a set of subjects whose value of the filter function ranges from 6.53 to 7.96, and the second node index is a set of subjects whose value of the filter function ranges from 6.75 to 8.18, and so on.

Table C. The effects of the phenotypic information to the magnitude of the disease component

| Variable | NYU dataset | | | PU dataset | | |
| --- | --- | --- | --- | --- | --- | --- |
|  | Missing data, *n* | F | P-value | Missing data, *n* | F | P-value |
| Age | 0 | 1.82 | 0.178 | 0 | 0.25 | 0.615 |
| Gender | 0 | 1.72 | 0.191 | 0 | 1.75 | 0.187 |
| Medication Status | 59 ADHD | 1.09 | 0.297 | 0 | 1.12 | 0.292 |

The statistical values were obtained after performing the general linear modeling with the magnitude of the disease component as a dependent variable and the group indicator as a dependent variable. Also, the phenotypic variable such as the age, gender or medication status as a covariate was included in the model, respectively.

Table D. The summary of receiver operating characteristic (ROC) analysis using the value of the filter function

| Parameters | NYU dataset | PU dataset |
| --- | --- | --- |
| Area under the ROC curve | 99.4 | 99.1 |
| Threshold value | 12 | 11 |
| Sensitivity, % | 98.3 | 96.3 |
| Specificity, % | 100 | 100 |
